# Supplementation with *Lactiplantibacillus plantarum* IMC 510 Modifies Microbiota Composition and Prevents Body Weight Gain Induced by Cafeteria Diet in Rats

**DOI:** 10.3390/ijms222011171

**Published:** 2021-10-16

**Authors:** Maria Vittoria Micioni Di Bonaventura, Maria Magdalena Coman, Daniele Tomassoni, Emanuela Micioni Di Bonaventura, Luca Botticelli, Maria Gabriella Gabrielli, Gian Maria Rossolini, Vincenzo Di Pilato, Cinzia Cecchini, Amedeo Amedei, Stefania Silvi, Maria Cristina Verdenelli, Carlo Cifani

**Affiliations:** 1 Pharmacology Unit, School of Pharmacy, University of Camerino, 62032 Camerino, Italy; mariavittoria.micioni@unicam.it (M.V.M.D.B.); emanuela.micioni@unicam.it (E.M.D.B.); luca.botticelli@unicam.it (L.B.); carlo.cifani@unicam.it (C.C.); 2 Synbiotec S.r.l., Spin-off of UNICAM, Via Gentile III Da Varano, 62032 Camerino, Italy; magda.coman@unicam.it (M.M.C.); cinzia.cecchini@unicam.it (C.C.); cristina.verdenelli@unicam.it (M.C.V.); 3 School of Biosciences and Veterinary Medicine, University of Camerino, 62032 Camerino, Italy; daniele.tomassoni@unicam.it (D.T.); gabriella.gabrielli@unicam.it (M.G.G.); 4 Department of Experimental and Clinical Medicine, University of Florence, 50134 Florence, Italy; gianmaria.rossolini@unifi.it (G.M.R.); amedeo.amedei@unifi.it (A.A.); 5 Microbiology and Virology Unit, Florence Careggi University Hospital, 50134 Florence, Italy; 6 Department of Surgical Sciences and Integrated Diagnostics, University of Genoa, 16132 Genova, Italy; vincenzo.dipilato@unige.it

**Keywords:** microbiota, 16S, obesity, cafeteria (CAF) diet, *Lactiplantibacillus (L.) plantarum* IMC 510, leptin, body weight, food intake

## Abstract

Changes in functionality and composition of gut microbiota (GM) have been associated and may contribute to the development and maintenance of obesity and related diseases. The aim of our study was to investigate for the first time the impact of *Lactiplantibacillus (L.) plantarum* IMC 510 in a rat model of diet-induced obesity, specifically in the cafeteria (CAF) diet. This diet provides a strong motivation to voluntary overeat, due to the palatability and variety of selected energy-dense foods. The oral administration for 84 days of this probiotic strain, added to the CAF diet, decreased food intake and body weight gain. Accordingly, it ameliorated body mass index, liver and white adipose tissue weight, hepatic lipid accumulation, adipocyte size, serum parameters, including glycemia and low-density lipoprotein levels, in CAF fed rats, potentially through leptin control. In this scenario, *L. plantarum* IMC 510 showed also beneficial effects on GM, limiting the microbial imbalance established by long exposure to CAF diet and preserving the proportion of different bacterial taxa. Further research is necessary to better elucidate the relationship between GM and overweight and then the mechanism of action by which *L. plantarum* IMC 510 modifies weight. However, these promising results prompt a clear advantage of probiotic supplementation and identify a new potential probiotic as a novel and safe therapeutic approach in obesity prevention and management.

## 1. Introduction

Obesity is a pandemic chronic disease associated with different co-morbidities, including diabetes, cardiovascular diseases, dyslipidemia and cancer [1], that is constantly expanding due to the westernization of diet and lifestyle [2,3]. A recent systematic study [4] indicated that obesity prevalence was doubled since 1980 in more than 70 countries, with approximately 107 million children and 603 million adults obese in 2015. According to the World Health Organization (WHO), obese subjects are those with a body mass index (BMI) higher or equal to the value 30 [5]. 

Obesity and related metabolic disturbances are related to changes of appetite modulators, including leptin [6], that might contribute to the dysregulation of food intake and, consequently, fat accumulation and energy homeostasis [7,8,9,10,11], resulting from the imbalance between excessive caloric intake compared to energy expenditure. In addition, other conditions may contribute to body weight gain, including genetic, epigenetic, metabolic, behavioral, environmental and cultural influences, as summarized in different reviews (i.e., [12,13,14,15,16]). Several promising approaches have been proposed for obesity treatment [17]: pharmacotherapy [18], bariatric surgery [19] and lifestyle modifications [20], but unfortunately limited weight loss was observed in the majority of the patients, particularly in children [21]. Thus, the etiology, the development and the management of obesity are really complex and multifactorial. Currently, an increasing interest has been reserved to the gut microbiota (GM), primarily on how its alterations could be involved in the vulnerability of this widespread disease [22,23] and the possibility of using probiotics as a potential therapeutic tool for weight control [24,25]. Many studies reported the successful use of probiotics, like *Lactobacillus*, *Bifidobacterium* and *Akkermansia*, to mitigate several metabolic disorders such as overweight, obesity, type 2 diabetes, hypertension and hypercholesterolemia [26,27,28]. To date, gut microorganisms showed the ability to decrease body weight, leptin levels, abdominal and epididymal fat volume, to down-regulate lipogenic genes and even to counteract *Enterobacter*-induced obesity (*Enterobacter cloacae* B29) and further, to restore the beneficial proportion of different bacterial taxa [29,30,31,32,33,34]. Interestingly, Ley and collaborators observed alterations in the distal intestinal microbiota of genetically obese mice compared to lean and wild-type siblings, presenting a major epididymal fat-pad mass to total body mass, with a specific significant increase in *Firmicutes* and a 50% reduction in *Bacteroidetes* [35]. As in humans, the two most detected bacterial phyla in rodents are the *Firmicutes* and the *Bacteroidetes*, characterized by a high ratio of *Bacteroidetes* to *Firmicutes* in normal-weight animals, while the opposite condition is found after obesity development [30,35,36]. However, the GM is densely populated by numerous microorganisms and its composition might be affected by several factors [37,38]. A comparison between obese and control subjects, regarding the abundance of *Lactobacillus* species, revealed higher levels of *Lactobacillus paracasei* and *Lactobacillus plantarum* in the latters, while *Lactobacillus reuteri* was highly represented in the obese subjects [39]. Overall, these findings highlight the pivotal role that several microorganisms may have in shaping gut homeostasis and how dietary intake can influence the composition of the microbiota.

For instance, the consumption of highly caloric and nutritionally poor foods has not only an impact on GM but also on leptin resistance [40,41]. This leads to a long-term loss of bacterial functions and mediates epigenetic changes in metabolic activities due to the Western diet, characterized by ultra-processed palatable caloric dense foods and sucrose-containing soft drinks [42,43,44,45].

Altogether, this knowledge prompts the research to better explore benefits for the health of the host, especially focusing on various species and strains of *Lactobacilli*. 

The aim of our study was to investigate, for the first time, the effect of 12-week supplementation with the *Lactiplantibacillus* (*L.) plantarum* IMC 510 in rats fed with cafeteria (CAF) diet compared to rats fed with standard food (CHOW diet). To address this issue, the composition of the GM was determined and compared to other factors, in order to evaluate how the CAF diet, with or without oral supplementation of the probiotic, affects body weight, food intake, leptin level, adipose tissue, liver and blood parameters.

This strong diet-induced obesity was chosen for its ability to promote weight gain [46,47] and the greater dysbiosis in rodents [48], providing ad libitum energy-dense human foods, similar to Western dietary habits [47,49]. The variety of food items includes appetible and calorically fat items that progressively lead to a higher motivation to overconsume them, and consequently facilitate an obesity state. 

The strain *L. plantarum* IMC 510 has been isolated and characterized for its probiotic properties by Synbiotec Srl (Camerino, Italy) [50,51,52]. 

## 2. Results

### 2.1. Effects of L. plantarum IMC 510-Dietary Supplementation on Body Weight and Feeding Consumption

**Figure 1:**
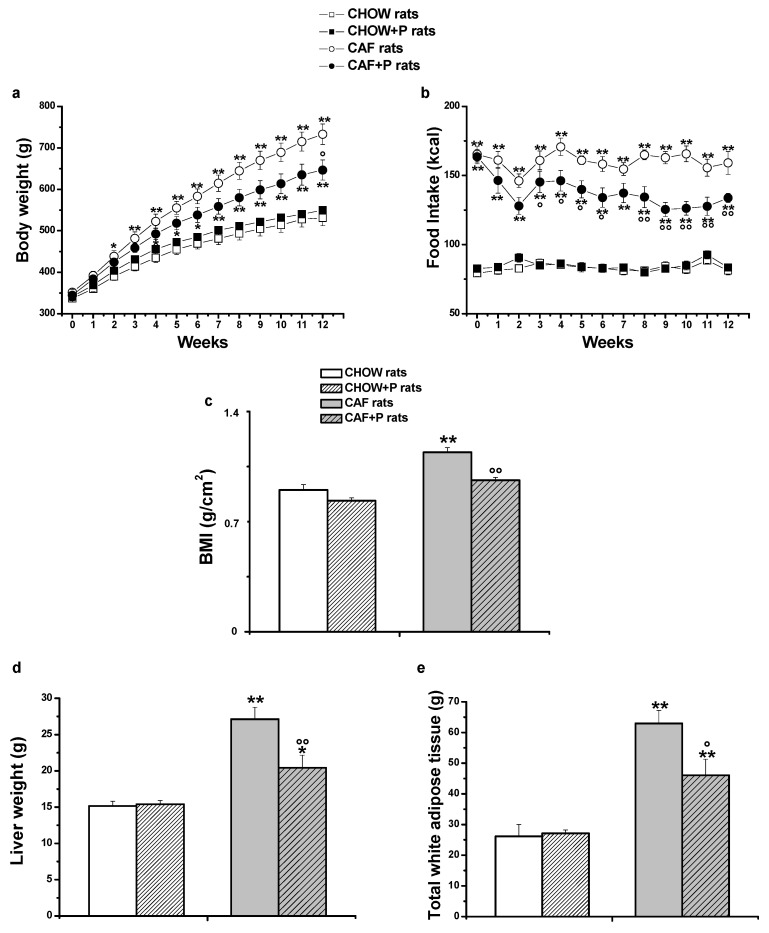
Body weight (g) (**a**) and Food Intake (kcal) (**b**) of male rats were measured daily throughout the experimental period. BMI (g/cm^2^) (**c**), liver (g) (**d**) and total white adipose (g) tissue (**e**) weight at day 84. Data are shown as means ± SEM of 6–9 rats for each group. Two-way ANOVA and Bonferroni test: * *p* < 0.05, ** *p* < 0.01 vs. CHOW rats; ° *p* < 0.05, °° *p* < 0.01 vs. CAF rats. CAF: cafeteria; P: probiotic supplementation; BMI: body mass index.

Figure 1a shows an upward trend in body weight in CAF rats. Specifically, two-way ANOVA (analysis of variance), which included the between-subject factors of diet (CHOW or CAF diet) and probiotic supplementation (no or yes), showed a significant interaction between these two factors [F (1, 27) = 5.37, *p* < 0.05]. Post-hoc comparisons indicated that CAF rats after 2 weeks and CAF rats treated with *L. plantarum* IMC 510 probiotic (CAF+P rats) after 4 weeks of CAF diet significantly increased their body weight compared to CHOW rats (*p* < 0.05). However, CAF+P rats showed body weight gain attenuated throughout the course of the study. 

In fact, at the end of the study, at day 84, the Bonferroni test revealed that both CAF rats weighed significantly more compared to CHOW rats (*p* < 0.01); however, the CAF+P weighed significantly 12% less with respect to the CAF counterparts (*p* < 0.01, Figure 1a).

Regarding food intake, two-way ANOVA showed a significant interaction between the two factors [F (1, 27) = 18.79, *p* < 0.01]. Post-hoc comparisons showed a significant increase in kcals assumed by both CAF rats compared to CHOW rats since the first day, but they also showed a clear trend of reduction in food intake in CAF+P versus CAF rats (Figure 1b).

As shown in Figure 1c, two-way ANOVA for the BMI at day 84 revealed a significant interaction between the two factors [F (1, 28) = 4.23, *p* < 0.05]. The Bonferroni test revealed that the BMI of CAF rats is significantly higher in comparison to both CHOW rats (*p* < 0.01) and CAF+P (*p* < 0.01).

As expected, liver [F (1, 28) = 8.6, *p* < 0.01] and total white adipose tissue weights [F (1, 28) = 5.3, *p* < 0.05] were significantly higher in CAF than in CHOW rats. In contrast, the probiotic supplementation significantly decreased the weight of liver (*p* < 0.01, Figure 1d) and the fat accumulation (*p* < 0.05, Figure 1e).

### 2.2. Effects of L. plantarum IMC 510-Dietary Supplementation on Blood Parameters 

A significant increase in glycemia (*p* < 0.01) and low-density lipoprotein (LDL, *p* < 0.01) level was found in CAF rats compared to CHOW rats, whereas the treatments significantly reduced them (*p* < 0.05) in CAF+P rats, as shown in Table 1. Moreover, we observed a strong trend toward a reduction of cholesterol, triglycerides and markers of liver disease levels in CAF+P rats compared to CAF rats.

No significant change in the leptin concentration was observed in the CHOW rats supplemented with the probiotics, as reported in Table 2. Instead, the leptin concentration significantly increased in the CAF rats compared to the CHOW groups, and conversely decreased in the CAF+P rats (*p* < 0.01).

### 2.3. Liver and Adipose Tissue: Morphological Analysis 

**Figure 2:**
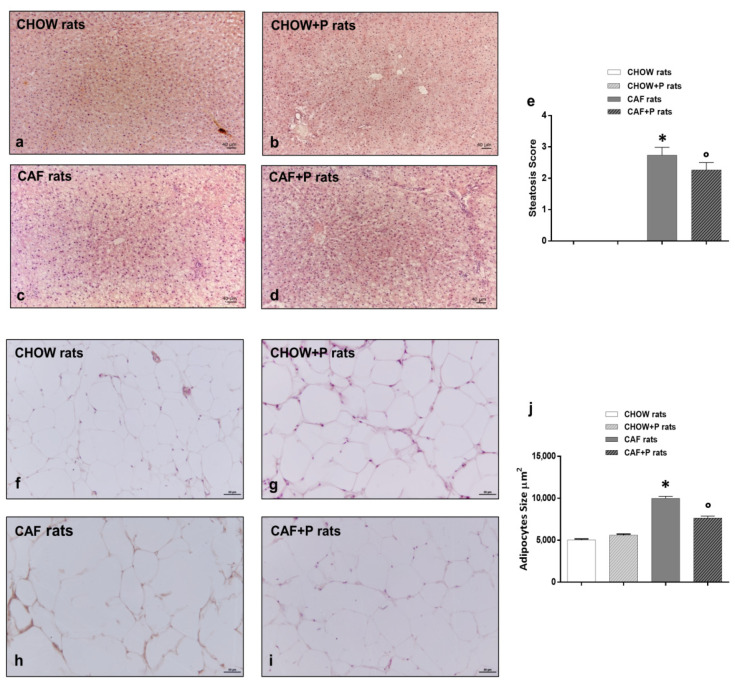
Hematoxylin and eosin (H&E) staining enhances the morphological features of liver (**a**–**d**) and visceral adipose tissue (**f**–**i**) of CHOW rats (**a**,**f**), CHOW+P rats (**b**,**g**), CAF rats (**c**,**h**) and CAF+P rats (**d**,**i**). The pictures of liver show normal structural organization and features, both for CHOW (**a**) and CHOW+P rats (**b**). On the contrary, liver sections from CAF rats show serious damages: largely extended areas of steatosis can be appreciated as well as the occurrence of inflammatory infiltrations. In CAF+P rats (**d**), the addition of the probiotic reduces the severity and extension of the steatotic condition. (**e**): the graph bar shows the score of steatosis, expressed as mean ± SEM. * *p* < 0.05 vs. CHOW, ° *p* < 0.05 vs. CAF. The pictures show an increase in the size of adipocytes area in CAF rats (**h**) compared to the CHOW (**f**) and CHOW+P (**g**) rats. The hypertrophy was counteracted in the CAF+P (**i**) rats. (**j**): the graph bar shows the size of adipocytes as mean ± SEM. * *p* < 0.05 vs. CHOW, ° *p* < 0.05 vs. CAF. Calibration bar (**a**–**d**): 40 µm. Calibration bar (**f**–**i**): 50 µm. CAF: cafeteria; P: probiotic supplementation.

Routine staining methods revealed different grades of damage in the liver parenchyma of the experimental groups examined, compared with the control ones (Figure 2). No fibrotic infiltrations were pointed out by application of the Masson’s trichrome staining.

No structural and cellular alterations were observed in the liver of either the CHOW rats (Figure S1 and Figure 2a) or the CHOW+P rats (Figure 2b).

In CAF rats (Figure 2c,d), the free access to the high caloric diet produced the development of extensive microvesicular steatosis and scattered macrovesicular steatosis, with a preferential periportal localization (Figure S1). The visualization of small infiltrating lymphocytes was also frequent, sometimes scattered in the liver parenchyma, sometimes associated with the bile ducts in the portal areas (Figure S1). The serious alteration of the parenchymal structure of CAF rats was scored 2.7 ± 0.3 for steatosis, compared to the CHOW liver scored as 0 (Figure 2e).

Probiotic supplementation to the CAF diet did not fully prevent the liver damages when compared with the morphological pattern of CHOW rat liver. Indeed, some areas of the hepatic parenchyma of CAF+P rats were characterized by lipid deposition, as well as by scattered inflammatory infiltrations. However, the extension of the centrolobular zones with preserved hepatocytes appeared to be wider with respect to the CAF rats (Figure 2d and Figure S2), with a steatosis score of 2.2 ± 0.2 for CAF+P rats (Figure 2e). Visceral adipose tissue analysis further confirmed the adipogenic action of the CAF diet. An increase in the size of the adipocyte was evident in the CAF group compared with CHOW and CHOW+P (Figure 2j) (*p* < 0.05 vs. CAF) as showed in Figure 2f–i. Supplementation with *L. plantarum* IMC 510 significantly inhibits the hypertrophy of the adipocytes (Figure 2h). The measure of the areas showed a decrease in the mean value for the CAF+P compared to CAF (Figure 2j) (*p* < 0.05 vs. CAF), indicating the positive impact of the probiotic supplementation on the adipogenic effects induced by the diet.

### 2.4. Modulation of the Gut Microbial Composition after L. plantarum IMC 510 Administration

#### 2.4.1. Quantitative Real-Time PCR

**Figure 3:**
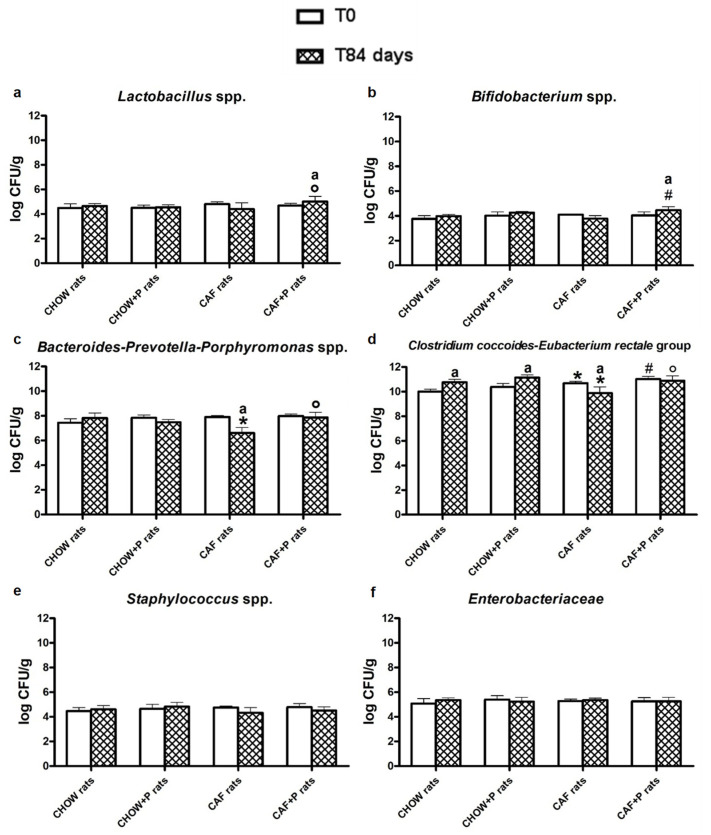
Fecal microbiota composition quantified by Real-Time PCR: (**a**) *Lactobacillus* spp.; (**b**) *Bifidobacterium* spp.; (**c**) Bacteroides-Prevotella-Porphyromonas spp.; (**d**) Clostridium coccoides-Eubacterium rectale group (**e**) Staphylococcus spp. and (**f**) Enterobacteriaceae in all groups of rats at the beginning of the experimental period (T0) and after 84 days of probiotic supplementation (T84). Data are shown as means ± SD of 6–9 rats per group. One-way ANOVA and Bonferroni test: * *p* < 0.05 vs. CHOW rats; ° *p* < 0.05 vs. CAF rats; ^#^ *p* < 0.05 vs. CAF+P rats; ^a^ *p* < 0.05 vs. T0. CAF: cafeteria; P: probiotic supplementation.

After 84 days, the CAF diet caused a significant decrease in *Bacteroides-Prevotella-Porphyromonas* spp. (*p* < 0.05) in the rats of this group related to the obesity status, while the probiotic supplementation balanced this level and significantly increased the concentration of *Lactobacillus* spp. and *Bifidobacterium* spp. in CAF+P rats (Figure 3a–c, *p* < 0.05). A similar trend as *Bacteroides* was noticed also for the *Clostridium coccoides-Eubacterium rectale* group in the CAF+P rats with respect to CAF rats (Figure 3d). No significant modification was found for the *Staphylococcus* spp. and *Enterobacteriaceae* among the rats’ groups during the experimental period (Figure 3e,f).

#### 2.4.2. Profiling of the GM by 16S Next-Generation Sequencing (NGS)

High-resolution genotyping of the fecal microbiota was performed to comprehensively characterize the gut microbial composition in all CAF rats, including the CAF-T0, CAF+P-T0, CAF-T84, CAF+P-T84 subgroups.

**Figure 4:**
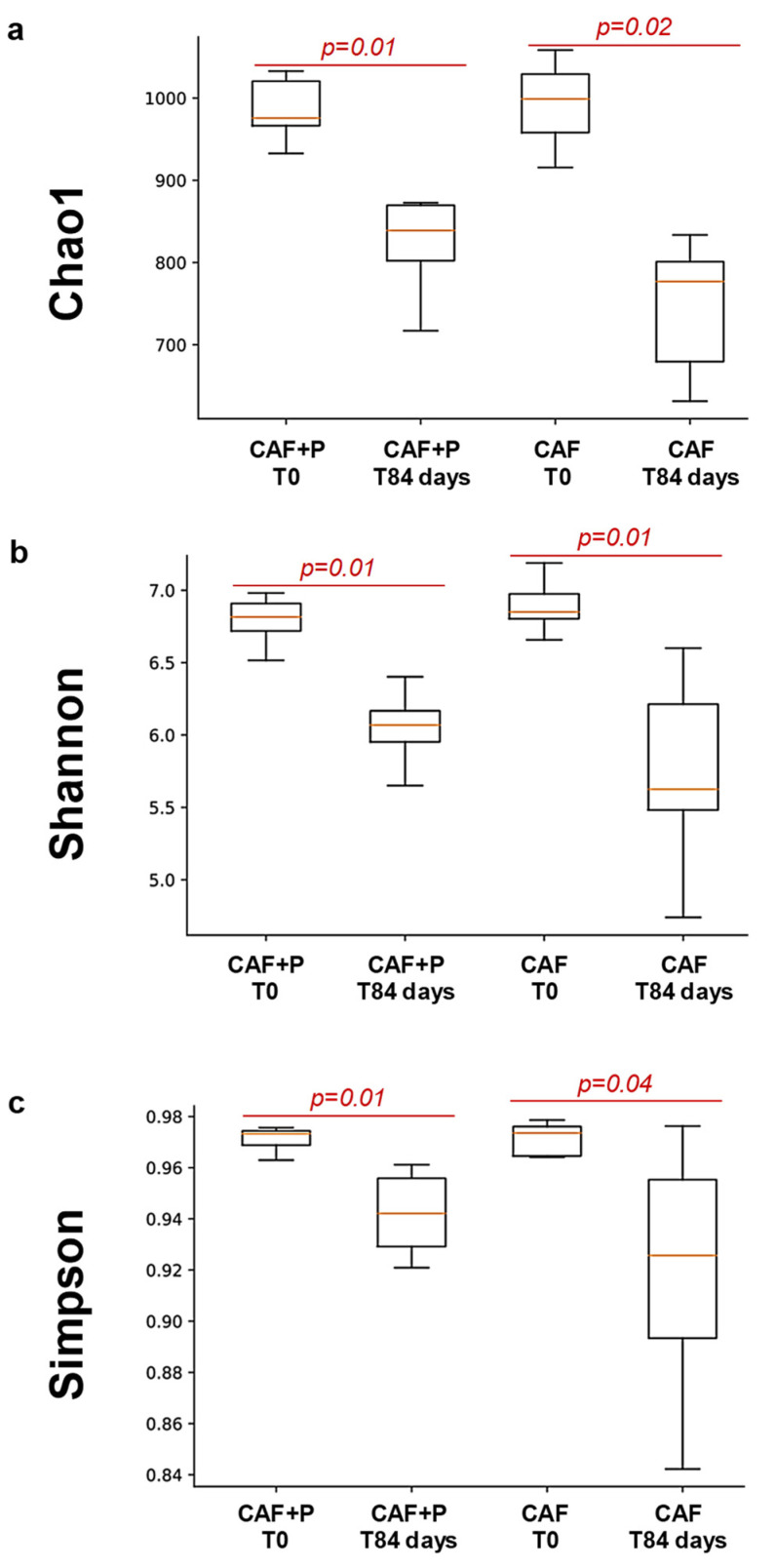
Comparison of alpha-diversity indices (**a**) Chao1, (**b**) Shannon and (**c**) Simpson in fecal samples collected at T0 and T84, within both CAF and CAF+P groups. Data are shown as means ± SD of 6–8 rats per group. *p* < 0.05 means significant differences in diversity indices (T84 vs. T0). CAF: cafeteria; P: probiotic supplementation.

An evaluation of alpha-diversity in fecal samples collected at T0 and T84, within both CAF and CAF+P groups, revealed significant differences (*p* = 0.01) in diversity indices (i.e., chao1, Shannon, Simpson) only according to the sampling time (i.e., T0 vs. T84) within each group, but not between groups (Figure 4a–c). Higher microbial diversity was consistently observed in CAF-T0 and CAF+P-T0 subgroups compared to their corresponding points at T84, suggesting that the CAF diet was a strong modifying factor of the GM composition, regardless of the probiotic supplementation. 

**Figure 5:**
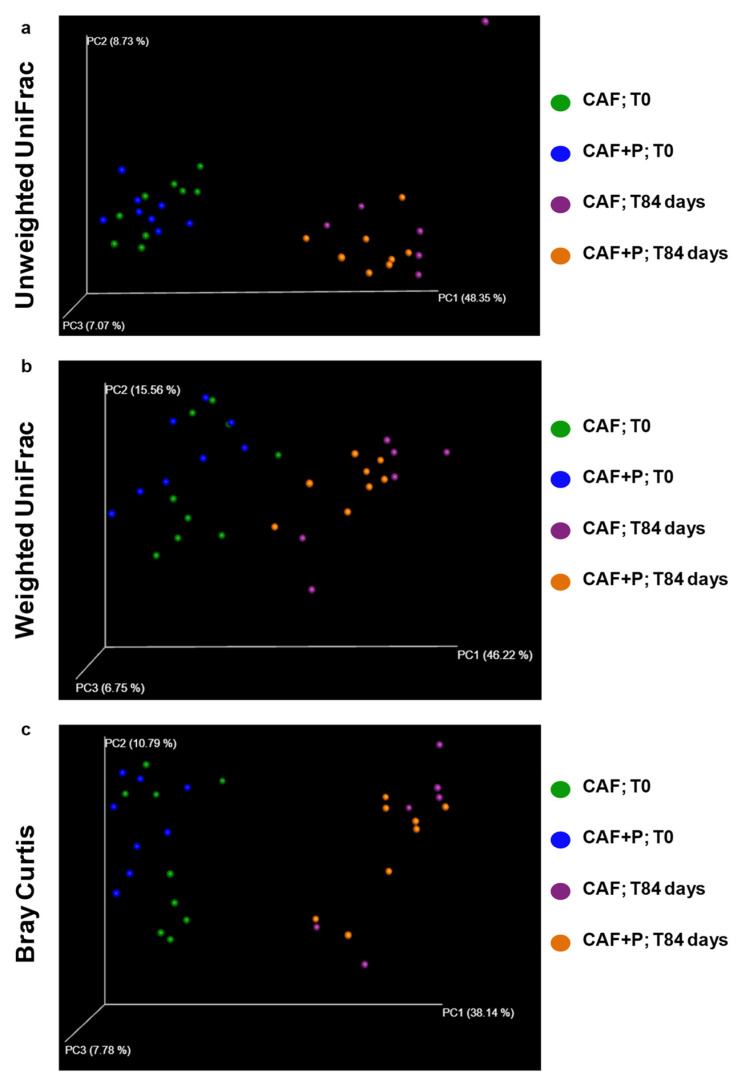
Principal Coordinates Analysis (PCoA) 3D plots. PCoA was performed using Unweighted UniFrac (**a**), Weighted UniFrac (**b**) and Bray-Curtis dissimilarity (**c**) distance matrices. Each sample is represented by a green point for CAF rats at T0, blue point for CAF+P group at T0, violet point for CAF group at T84 days and orange point for CAF+P group at T84 days. CAF: cafeteria; P: probiotic supplementation.

Consistently, evaluation of beta-diversity through Principal Coordinates Analysis (PCoA) using different metrics (i.e., Bray-Curtis, weighted and unweighted UniFrac) showed that samples from the CAF-T84 and CAF+P-T84 groups were uniformly distributed within a single cluster, indicating no substantial differences in the microbiota structure of these samples and clustered away from the corresponding control groups, CAF-T0 and CAF+P-T0 (Figure 5a–c). As before, significant differences (PERMANOVA, *p* = 0.03) in beta-diversity metrics were observed only within each subgroup of CAF and CAF+P (i.e., CAF-T0 vs. CAF-T84 and CAF+P-T0 vs. CAF+P-T84).

**Figure 6:**
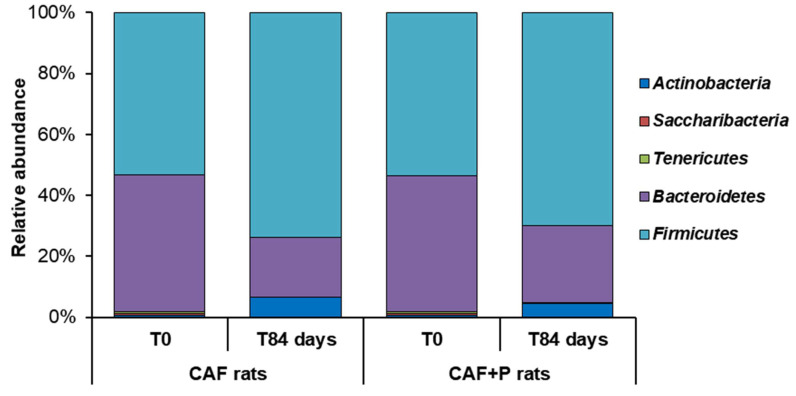
Modulation of microbiota community by *Lactiplantibacillus plantarum* IMC 510. Relative abundance of bacterial phyla differing between the groups, fed on CAF diet supplemented or not with probiotics. CAF: cafeteria; P: probiotic supplementation.

Analysis of the taxonomic composition at baseline revealed the presence of five major bacterial phyla: *Firmicutes*, *Bacteroidetes*, *Actinobacteria*, *Tenericutes* and *Saccharibacteria*, which accounted for about 90% of the total composition in each subgroup; among these, *Firmicutes* and *Bacteroidetes* were the most abundant members (Figure 6). No significant differences in the taxonomic composition between the CAF and CAF+P groups were identified at T0. The same major phyla were identified in samples belonging to CAF-T84 and CAF+P-T84 subgroups, although with different proportions compared to the corresponding baseline (Figure 6). Interestingly, similar taxonomic variation trends were prospectively identified within the CAF and CAF+P groups from T0 to T84, with a significant increase in *Firmicutes* (from 48% to 64% in CAF, *p* = 0.01 and from 50% to 63% in CAF+P, *p* = 0.02) and a concomitant decrease in *Bacteroidetes* (from 40% to 17% in CAF, *p* < 0.01 and from 41% to 23% in CAF+P, *p* = 0.01).

**Figure 7:**
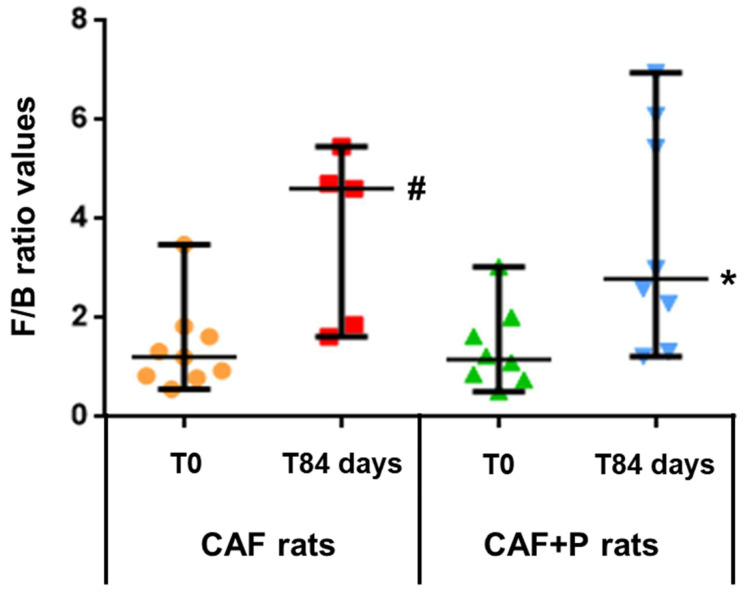
*Firmicutes/Bacteroidetes* (F/B) ratio calculated at T0 and after 84 days of CAF diet without and with probiotic supplementation. ^#^ *p* < 0.05 vs. CAF rats T0; * *p* < 0.05 vs. CAF rats T84. CAF: cafeteria; P: probiotic supplementation.

Overall, the CAF diet-induced obesity was associated with an increased *Firmicutes/Bacteroidetes* (F/B) ratio in both CAF and CAF+P groups at T84, although with a different magnitude (Figure 7). Specifically, the F/B ratio was significantly higher in CAF than CAF+P rats at T84 with respect to T0 (*p* < 0.05), suggesting that the probiotic supplementation could have had a role in maintaining a significantly lower ratio. This hypothesis was also consistent with previous results obtained by quantitative Real-Time PCR experiments.

**Figure 8:**
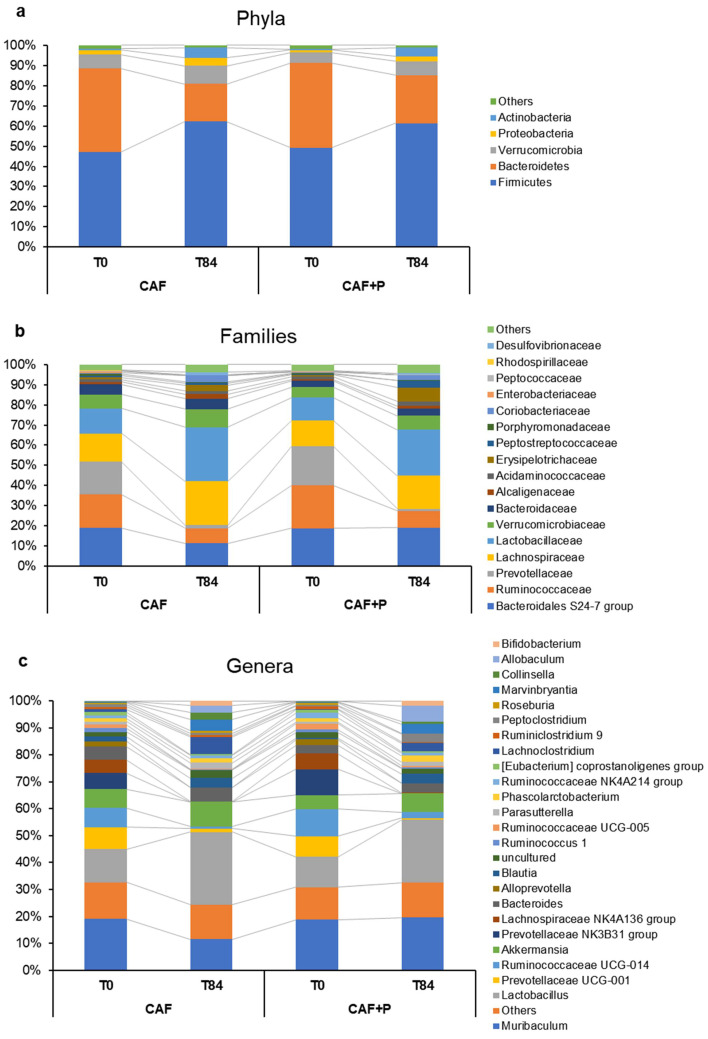
Histogram of relative abundance. The x-axis represents groups (CAF and CAF+P) and sampling times (T0 and T84 days) and the y-axis represents relative abundance presented as percentage. (**a**) Relative abundance of the top 5 phyla; (**b**) Relative abundance of the top 17 families; (**c**) Relative abundance of the top 25 genera; other species were combined as “Others”. CAF: cafeteria; P: probiotic supplementation.

Interestingly, the F/B ratio was found to be influenced more by the decrease in *Bacteroidetes* than by the increase in *Firmicutes* over time, primarily in CAF rats. While the abundance of *Bacteroidetes* remained substantially unchanged (around 18%) in the CAF+P group, it markedly decreased (18 vs. 10%, *p* < 0.01) from T0 to T84 in the CAF group (Figure 8a,b). Within the *Bacteroidetes* phylum, the *Bacteroidales* S24-7 was among the most represented bacterial families and a peculiar variation of its relative abundance was observed within the study period (Figure 8b). 

At the genus level, several of the most represented bacterial genera at T0 were also detected at T84 in the CAF and CAF+P groups, but with significant changes in their relative abundances: groups such as *Prevotellaceae* UCG-001, *Ruminococcaceae* UCG-014, *Prevotellaceae* NK3B31 group, *Lachnospiraceae* NK4A136 group, *Alloprevotella*, *Ruminococcus* 1, *Ruminococcaceae* UCG-005 were the most abundant at T0, but all of them significantly decreased at T84 (with or without probiotics supplementation) (Figure 8c). 

After the exposure to the CAF diets, *Lactobacillus*, *Muribaculum*, *Akkermansia*, *Lachnoclostridium*, *Bacteroides*, *Marvinbryantia* and *Blautia* were the most represented genera. Among these, the genus *Muribaculum* (belonging to the *Bacteroidales* S24-7 family group) was highly represented in all subgroups except CAF-T84, the most abundant genus, and strongly influenced the F/B variations observed among the CAF and CAF+P groups during the study period (Figure 8c). 

A correlation analysis was also carried out to investigate potential relationships between the abundance of bacterial taxa and the variation in body weight and in food intake observed between T84 and T0. Overall, different bacterial genera, mainly belonging to the *Ruminococcaceae* and *Lachnospiraceae* families, were found to be positively or negatively correlated with the body weight gain (expressed as the difference in body weight at T84-T0) and the net food-intake (expressed as the difference in food-intake at T84-T0) (Table 3), suggesting different effects of *L. plantarum* IMC 510 supplementation.

#### 2.4.3. Intestinal Colonization of *L*. *plantarum* IMC 510

Fecal samples from the CAF+P rats were analyzed at time 0 and after 84 days of *L. plantarum* IMC 510 supplementation. In the collected fecal samples, the initial (T0) amount of lactobacilli was 5.01 × 10^4^ CFU/g, while after the probiotic treatment the cell count increased, even if non-significantly (*p* > 0.05), reaching 1.32 × 10^5^ CFU/g of feces. The probiotic strain, *L. plantarum* IMC 510, has been detected from fecal samples of the CAF+P rats, but not from those of the control group (CAF rats). At the end of the consumption period (T84) the average percentage of the recovery of *L. plantarum* IMC 510 was positive on all subjects from the CAF+P group (Table 4).

## 3. Discussion

In our study of male rats, long-term exposure to CAF diet increased caloric food intake, body weight, also eliciting changes in the GM composition (microbial imbalance) and blood parameter levels, including glycemia and leptin. In accordance with the body weight gain results, also BMI, liver and white adipose tissue weight were significantly higher in CAF-fed rats. This diet was chosen and preferred to other diets inducing obesity in animal models, because it provides a strong motivational stimulus to voluntarily overeat, thanks to the palatability and variety of selected energy-dense foods, reflecting the CAF diet in modern society [47,49]. In addition to obesity, it also induces glucose intolerance and generalized inflammation [46] and severe dysbiosis [48], similar to some conditions of Western diet-associated diseases in overweight and obese subjects. Here, the CAF diet allows studying the diet-induced microbial imbalance and, in turn, evaluating for the first time the effect of *L. plantarum* IMC 510 in line with metabolic changes observed in humans. 

This probiotic strain was isolated from healthy elderly subjects, with a high capacity to adhere to the intestinal mucosa, confirming excellent gut colonization that promotes a high probiotic properties expression [51,52]. The choice of this probiotic, together with the selection of the CAF diet, represent a novel approach in the source of this probiotic strain compared to those used in the already published papers, in which the *Lactobacilli* have food origins or similar (sourdough, such as in [53]). The human origin gave also better chances in expressing beneficial effects in humans.

The oral and daily supplementation of *L. plantarum* IMC 510 for 84 days significantly attenuated food intake; therefore, the body weight gain and other parameters (discussed below) in rats under the CAF diet and showed beneficial effects on GM, eliminating the microbial imbalance established by the obesity status and restoring the microbiota equilibrium. The decreased dietary intake could be associated with alterations in the intestinal flora in response to *L. plantarum* IMC 510 administration. Indeed, microbes could affect the food preference modifying the receptor expression or transduction. Variations in the activity and expression of taste receptors were reported following gastric bypass surgery, which also impacted GM, satiety and food preferences [54]. The conflict between host and microbiota influences craving for selective nutrients, and additionally, it is supposed to affect satiety and caloric consumption [55,56]. The CAF diet provides an extra energy supply which leads to a reduction of GM diversity, triggering a mechanism that could promote protracted alterations in satiety, damaging the host and inducing obesity. Thus, the treatment with probiotics enhanced the GM diversity, which alters the satiety setpoint and improves the decrease in food intake of the host [55].

Moreover, the recovered equilibrium of the microbiota is crucial to maintain stable gut permeability and Lipopolysaccharide (LPS) absorption, which dramatically increase following a high-fat diet (HFD) and elicit low-grade inflammation, metabolic disorders and the development of insulin resistance [57,58]. The LPS content represents a key factor in the development of inflammation in obese conditions related to GM dysbiosis and *L. plantarum* IMC 510 already showed strong antipathogenic activity against Gram-positive and Gram-negative bacteria [50].

*Firmicutes* and *Bacteroidetes*, the two key bacterial phyla of the human GM, have been demonstrated to regulate the energy homeostasis in obesity and in particular, a higher F/B ratio is detected in obese subjects, highlighting the relevant role of GM in the fat metabolism regulation [59]. 

Our results revealed a significant increase in the relative abundance of *Firmicutes* and a higher F/B ratio in obese rats (CAF rats), in line with other works [26,30,31], while the F/B ratio value significantly decreased in the probiotic supplemented CAF rats (CAF+P rats). Therefore, the probiotic administration maintains a lower F/B ratio despite the CAF diet consumption, extending previously obtained results [26,30,31] and reinforcing the probiotics’ use in obesity management. 

The major contribution to the F/B ratio variations observed among the CAF and CAF+P groups during the experimental period was made by different proportions of the genus *Muribaculum*. The *Muribaculum* genus, belonging to *Bacteroidales* S24-7, is normally present in the healthy mouse gut microbiome [60], and it is known to decrease in mice during the consumption of the Western diet [61]; furthermore, *Muribaculum* is associated with the regulation of body weight and carbohydrate metabolism [60,62]. Interestingly, the probiotic supplementation could alleviate the decrease in this important species of bacteria and the family of bacteria it belongs to, influencing in a positive way the amount of energy available to its host. At the family’s level, an increase in *Lachnospiraceae* was observed in both CAF and CAF+P rats after 84 days of dietary intervention, but to a lesser extent in the CAF+P group. *Lachnospiraceae* bacteria (phylum *Firmicutes*, class *Clostridia*) have also been linked to obesity [63]. In addition, a metagenomic study indicated that the taxonomic family *Lachnospiraceae* may also be specifically associated with type 2 diabetes in both humans and mice models [64]. The *Lachnospiraceae* family has been shown to have a positive correlation with inflammation markers in white adipose tissues and body weight gain in diet-induced obese mice [65]. Supplementation with *L. plantarum* IMC 510 has been shown to alleviate the increase in *Lechnospiraceae* during an HFD and so, it may be involved in the reduction of obesity-related inflammatory status. 

Again, at the family level, the present study highlighted an increase in *Erysipelotrichaceae* in the CAF+P group after 84 days of dietary treatment. Interestingly, members of this family have been shown, in several independent studies, to differentiate in abundance, in response to changes in the amount of dietary fat intake [57]. In our study, the genus *Allobaculum*, a member of the family *Erysipelotrichaceae*, increased in the CAF+P group after 84 days. Previous work has shown that low-fat feeding was associated with an increase in the genus *Allobaculum* compared with HFD feeding [65]. Moreover, treatment with the plant alkaloid berberine, which prevents obesity and insulin resistance in rats fed an HFD, increased the abundance of *Allobaculum* [66]. 

The same result was obtained with the probiotic supplementation in our study, confirming the hypothesis of a potential benefit of this bacterial genus for the physiology of the host.

We found no difference in the GM of rats under standard diet, treated with and without the probiotic, according to the limited evidence for the effect of probiotic supplementation when the GM is unperturbed by pathophysiological conditions or treatment, including antibiotics or chemotherapy [67,68].

Assessing different serum parameters, CAF diet caused hyperglycemia and affected serum lipid profile as well as GOT, GPT and GGT compared to a standard diet. Regarding cholesterol level, we found a strong increase in its concentration, even though not reaching statistical significance in CAF rats. It is well known that rodents are resistant to develop hypercholesterolemia [69,70,71,72], unless longer exposure to HFD or specific cholesterol-enriched diets [73,74]. However, the probiotic supplementation showed a clear trend toward a reduction of cholesterol, triglycerides and a significant decrease in glycemia and LDL levels in CAF+P rats. 

Notably, the supplementation with *L. plantarum* IMC 510 proved to be associated with the decrease in serum leptin levels compared to the high levels induced by the CAF diet, consistent with its ability to reduce body weight gain and fat mass accumulation. Previous studies indicated that probiotics intervention decreased circulating leptin levels [31,32,72,75]. In addition, Yao and co-authors recently found that GM absence can affect body weight and leptin level, reporting that a GM depletion increased body weight, plasma leptin level and leptin expression by epigenetic modulation (DNA methylation), with a high risk of leptin resistance [76]. Remarkably, the elevated circulating leptin is considered a biomarker of leptin resistance, which is commonly detected in obese compared to non-obese subjects, reflecting that an increased calorie-dense food intake that can lead to hyperphagia and thus difficulty in losing body weight [6,77,78]. In fact, leptin, a potent anorexigenic hormone secreted from adipocytes, binding to specific receptors in the central nervous system, primarily in the hypothalamus [79], modulates satiety, metabolism, energy balance, body weight homeostasis and neuroendocrine response [79,80,81]. Then, the disruption of leptin signaling may contribute to the development of metabolic complications, including diabetes and cardiovascular diseases [82]. It is remarkable the positive effects of *L. plantarum* IMC 510 supplementation in restoring leptin levels and mitigate leptin resistance.

In line with this, we observed in CAF+P rats a significant reduction of white fat accumulation and adipocyte hypertrophy upon probiotic supplementation, compared to CAF rats. These effects are significant for the potential anti-obesity action of *L. plantarum* IMC 510, considering that excessive fat accumulation and hypertrophy represent relevant markers for obesity conditions [83,84].

Histological analysis of the rat liver revealed an increase in cellular lipid deposits in CAF rats, typical of steatotic pathologic change, and they were evidenced when compared with the CHOW group. Supplementation with *L. plantarum* IMC 510 seems to prevent this hepatic lipid accumulation, caused by diet, and the overall morphology was maintained with a lesser extent of steatotic area. This could be correlated to a decrease in body weight and visceral fat depots and could explain the low blood serum level of GOT and GPT in supplemented CAF rats. In fact, both inflammatory cytokines and free radicals generally induce damage to hepatocytes and have a critical role in the pathogenesis of HFD induced liver injury [85,86,87]. Notably, different studies [88,89,90] have attempted to develop specific probiotics to treat liver inflammation and decrease GOT and GPT serum levels. For instance, in humans, oral administration of probiotic capsules containing *L. acidophilus, L. bulgaricus, Bifidobacterium lactis* and *Streptococcus thermophilus* decreased the levels of GOT and GPT in the serum [91]. 

As a limitation of this work, further investigations will be necessary to study the potential protective role of *L. plantarum* IMC 510 on body weight gain and in the hepatic steatosis induced by CAF diet and to explain the possible molecular mechanisms underlying its correlation with leptin, the anti-inflammatory and antioxidant properties of probiotics’ supplementation [92,93,94]. Moreover, *L. plantarum* IMC 510 needs to be evaluated in a large cohort of Sprague-Dawley rats after the development of obesity-prone and obesity-resistant (OR) phenotypes. This allows the evaluation of its action on inter-individual susceptibility in weight gain in response to obesogenic diets. 

## 4. Materials and Methods

### 4.1. Subjects and Diet Composition

A total of 36 Male Sprague-Dawley rats (Charles River, Calco, Italy) were used. The body weight of the rats was 300–350 g at the beginning of the experiments. Rats were acclimated to individual cages under a 12-h light/dark cycle (lights on at 08:00 am) with *ad libitum* chow (4RF18, Mucedola, Settimo Milanese, Italy; 2.6 kcal/g) and water for 2 weeks prior to the experiments. They were kept in a room at constant temperature (20–22 °C) and humidity (45–55%). 

Since no significant differences in body weight (*p* > 0.05) and food intake (*p* > 0.05) were detected, rats were randomly divided into two experimental groups as follows (n = 18 in each group): animal fed with chow only, called CHOW rats and animal fed 24 h with both chow and extended access to CAF diet for 84 days, called CAF rats. The CAF diet, previously described [95,96], consisted of mortadella (3.2 kcal/g), cookies (Macine, Mulino Bianco; 4.8 kcal/g), chocolate muffin (Mr Day, Vicenzi group; 4.5 kcal/g), cheese chips (Fonzies; 5.3 kcal/g), cheese (Biraghi cheese, 4.2 kcal/g), sippets (San Carlo; 5.5 kcal/g) and lard (9 kcal/g), which were individually weighed before being made available to the rats. Each group was divided into two subgroups (n = 9 in each group): control groups (CHOW or CAF rats) and supplemented groups with *L. plantarum* IMC 510 probiotic strain (CHOW+P or CAF+P). Probiotic was dissolved in drinking water (10^8^ probiotic cells/die in 30 mL), daily prepared to prevent differences in viability, and as soon as rats consumed the entire probiotic solution, they had free access to the water.

Weight gain and caloric intake were recorded every day for 12 weeks. Caloric intake and macronutrient composition were calculated by weighing each kind of food before and after the meal, using the nutritional information provided by the manufacturer. At the end of the study, BMI was calculated (body weight (g) divided by the square of the anal–nasal length (cm^2^)) and, after sacrificing, liver and white adipose tissue were immediately excised and weighed.

### 4.2. Probiotic Supplementation

The strain *L. plantarum* IMC 510 was isolated from healthy elderly subjects during the European project Crownalife [97] and in vitro tested for all the probiotic characteristics: resistance to low pH, bile salts and pancreatic juice, ability to adhere to intestinal cells and colonize the mucosa, antipathogenic activity against bacteria (Gram + and Gram −) and yeasts, non-transmissible antibiotic resistance genes’ absence of plasmids [50,51,52].

This strain was produced by fermentation in a 30-L pilot fermenter (Pierre Guerin technologies, Mauzé-sur-le-Mignon, France) and lyophilized diluting the cells biomass in PBS solution at 10% glycerol (*w*/*v*) added at a ratio 1:5 to the biomass. The biomass was frozen at −80 °C for at least 30 min and then lyophilized using a Zirbus freeze dryer (ZirbusVaco 2, Bad Grund, Germany) with a condenser temperature of −50 °C and a chamber pressure *p* < 0.08 mbar for 48 h. After the lyophilization process, the probiotic powder was analyzed to determine the viable cell concentration (CFU/g). 

The probiotic strain *L. plantarum* IMC 510 was daily administered for 84 days, since the first day of access to the CAF diet, at a 10^8^ probiotic cells/die concentration, dissolving the lyophilized powder into the water drinking bottle of each single rat. Every morning the probiotic powder was dissolved in approximately 30 mL of water in a standard drinking bottle. As soon as the rats consumed the entire probiotic solution, a fresh bottle was provided in order to have free access to the water.

### 4.3. Blood Parameters 

Blood samples were collected in 1 mL L-heparin tubes (Sarstedt, Germany) and the serum was centrifuged at 3000 rpm for 10 min. They were stored at 4 °C and delivered to the Fioroni laboratory (San Benedetto del Tronto, AP, Italy) and analyzed within 24 h. The leptin concentration was evaluated by colorimetric method using a specific kit (Rat Leptin ELISA Kit, Abcam ab100773, Cambridge CB2 0AX, UK) following the protocol of the datasheet.

### 4.4. Liver and Adipose Tissue: Morphological Analysis

After the sacrifice, the liver and the visceral adipose tissue were removed and immediately immersed in Bouin’s fixative solution (picric acid, 4% formaldehyde and acetic acid in 0.1M PBS, pH 7.4) for 12 h at room temperature. After fixation, the samples were gradually dehydrated and routinely embedded in paraffin. Sections (5 µm thick) were cut and collected on Superfrost plus slides. The Hematoxylin-Eosin stain was used for the evaluation of tissue morphology. In the liver, Masson’s trichrome staining was applied to visualize collagen fibers.

Sections were viewed under a light microscope. The images were transferred from the microscope by DS-R12 NIKON camera and evaluated using a NIS Elements Nikon image analyzer. To validate the histological features and to determine the hepatic steatosis, a scoring system was applied [98]. Briefly, steatosis scores were defined as follows: score 0, presence of intrahepatic fat droplets in <5% of hepatocytes; score 1, presence of intrahepatic fat droplets in 5–33% of hepatocytes; score 2, presence of intrahepatic fat droplets in 33–66% of hepatocytes; and score 3, presence of intrahepatic fat droplets in >67% of hepatocytes. For the visceral adipose tissue, using a specific function of the program, the area of cells was measured.

### 4.5. Microbiota Composition Analysis of Fecal Samples

Fecal samples were collected from each rat from experimental groups (CHOW, CHOW+P, CAF and CAF+P rats) at time 0 (T0), corresponding to the starting day of probiotic supplementation, and after 84 days of probiotic supplementation (T84). The feces were frozen at −80 °C until performing the microbiota analysis (quantitative Real-Time PCR, 16S next-generation sequencing (NGS) analysis and probiotic colonization).

#### 4.5.1. Bacterial DNA Extraction

DNA extraction from all fecal samples was performed using a Stool DNA Isolation Kit (NorgenBiotek Corp., Thorold, Canada) with a modified protocol following the manufacturer’s instructions specific for the fecal samples. Quantity and purity of all extracted DNA were checked with NanoDrop ND-1000 Spectrophotometer (Thermo Fisher Scientific, Waltham, MA, USA) and then stored at −20 °C until used for molecular analysis.

#### 4.5.2. Quantitative Real-Time PCR

A quantitative Real-Time PCR (qPCR) procedure was used for the quantification of selected bacterial groups from CHOW, CHOW+P, CAF and CAF+P rats’ feces. The bacterial groups of interest were *Lactobacillus* spp., *Bifidobacterium* spp., *Bacteroides-Prevotella-Porphyromonas* spp., *Staphylococcus* spp., *Clostridium coccoides-Eubacterium rectale* group and *Enterobacteriaceae*. Specific primers were used and SYBR Green Quantitative Real-Time PCR amplification was performed using an iCycleriQ Real-Time Detection System (Stratagene) associated with MXP Software using the conditions and the standard curves for each bacterial group [99].

#### 4.5.3. 16S NGS and Analysis

The 16S metagenomic analysis was carried out using an NGS approach [100]. Briefly, total DNA extracts were used for PCR amplification of the V3–V4 variable regions of the bacterial universal gene coding for the 16S rRNA. The amplification products were processed for massive sequencing through the NGS Illumina MiSeq platform (Illumina Inc., San Diego, CA, USA) using a 2 × 300 bp paired-end approach. Sequenced reads were merged using PEAR [101] and processed with USEARCH 6.1 [102] to detect potential chimera sequences and to cluster merged amplicons in operational taxonomic units (OTUs), with a minimum pair-wise identity threshold of 97%. The SILVA database (release 128) was employed for taxonomic classification [103]. Evaluation of microbial alpha (Chao1, Simpson’s and Shannon’s diversity) and beta (UniFrac distances, Bray–Curtis dissimilarity) diversity measures were performed using QIIME (v. 1.9) [104]. 

#### 4.5.4. Recovery of *L*. *plantarum* IMC 510

To confirm the presence of the tested strain in the intestine, fecal samples of all rats were collected at T0 and at the end of 84 days of probiotic supplementation. Fecal samples were analyzed by enumeration of vancomycin and gentamicin-resistant lactobacilli onto modified-MRS agar by a 10-fold serial dilution method [52].

After aerobic incubation at 37 °C for 48–72 h, ten to twenty percent of the total colonies per sample randomly selected from countable agar plates were isolated and checked for purity. DNA extracted from the selected colonies using a modified benzyl chloride method [105] was analyzed by the RAPD technique [52].

### 4.6. Statistical Analysis

In the experiment, rats were allocated to experimental groups using a simple randomization approach; prior to allocation, potential differences in body weight and food intake were assessed.

In vivo and ex vivo results were presented as mean ± SEM. The results were expressed as mean ± standard deviation. 

In vivo data were analyzed by two-way ANOVA (Systat Software 10.0, San Jose, CA, USA) for repeated measures, when necessary, which included the between-subject factors of diet (CHOW or CAF diet) and probiotic supplementation (no or yes). We used post-hoc tests to follow up on significant interaction or main effects (*p* < 0.05) from the factorial ANOVAs. 

To avoid misinterpretation of the effect of the probiotic, rats that were resistant to increasing body weight [106,107,108,109] and thus developing the obese phenotype [110,111,112] were excluded from the experiment. As described above, the probiotic supplementation started on the first day of access to the CAF diet, and thus the phenotype was not yet clearly expressed. However, the early adaptations to a fat diet are crucial for obesity development or resistance and the first week of exposure is highly predictive of weight gain over the subsequent weeks [113,114]. For this reason, at the end of the first week of the CAF diet ad libitum, 4 rats (3 CAF and 1 CAF+P rats) were excluded from the study. They were potentially susceptible to develop obesity resistance, showing a weight gain significantly lower (g: OR-CAF rats 22.7 ± 1.2; OR-CAF+P rat 23.0) compared to the other rats under CAF diet (*p* < 0.05, g: CAF rats 40.2 ± 4.7; CAF+P rats 39.4 ± 3.5) and similarly to the control CHOW groups (g: CHOW rats 23.1 ± 2.0; CHOW+P rats 27.7 ± 2.8).

For ex vivo data, significant differences between mean values were determined by Bonferroni multiple comparison test after One-way ANOVA using GraphPad PRISM^®^ 5.1 (GraphPad Software, CA, USA). 

For 16S NGS, data were assessed for normality with the Shapiro–Wilk test using GraphPad Prism 6, and proper statistical analyses were performed by dedicated scripts implemented in QIIME, using: (i) the Kruskal–Wallis test to evaluate potential differences in the relative abundance of bacterial taxa on pairwise or multiple comparisons and to evaluate differences in alpha-diversity indices (i.e., Chao1, Simpson, Shannon); (ii) the permutational ANOVA (PERMANOVA) to test significance (999 permutations) between samples’ clusters generated by the PCoA, using different beta-diversity metrics (i.e., UniFrac, Bray–Curtis). Spearman correlation coefficients between relative abundances of microbial taxa and levels of body weight gain and food intake were computed using GraphPad Prism 6. Statistical significance was defined with a *p*-value less than 0.05.

## 5. Conclusions

Our data extend the investigation of the manipulation of GM that might represent a potential target for obesity management, evaluating the effects of a novel and specific probiotic strain in rats consuming Western-style foods, similar to the human diet. In fact, many findings revealed that certain probiotic strains could counteract overweight [22,23,24,25,33,59], while others produced mild effects or even lead to weight gain [57,115,116,117]. In the current study, for the first time, *L. plantarum* IMC 510 was tested, for 84 days, in male rats under the CAF diet. The probiotic preserved the major bacterial phyla in GM composition in the CAF+P group, compared to their CAF rats counterpart without supplementation. Moreover, *L. plantarum* IMC 510 was able to decrease the amount of food intake, weight gained and, consequently, relative beneficial effects were demonstrated by serological, biochemical and histological analyses, potentially through leptin control. Even though further research is needed to further elucidate this mechanism, our findings support the positive hypothesis that specific probiotic strains, such as *L. plantarum* IMC 510, can be considered as a promising therapeutic option to counteract and potentially prevent overweight, obesity and related comorbidities.

## Figures and Tables

**Table 1:** Blood parameters levels at the end of the experiment (day 84).

Blood Parameters (mg/dl)				
	CHOW Rats	CHOW+P Rats	CAF Rats	CAF+P Rats
Glycemia	90.8 ± 1.9	92.9 ± 3.8	110.8 ± 2.2 **	94.1 ± 3.5 °
GOT	154.5 ± 9.0	150.9 ± 9.0	169.0 ± 8.3	144.9 ± 6.9
GPT	25.6 ± 2.3	26.7 ± 1.8	29.0 ± 2.7	23.5 ± 2.8
GGT	0.4 ± 0.1	0.5 ± 0.1	0.7 ± 0.3	0.5 ± 0.1
Cholesterol	109.0 ± 5.4	105.7 ± 5.7	128.8 ± 8.9	113.1 ± 7.3
HDL	29.0 ± 1.6	28.7 ± 2.3	24.3 ± 1.2	26.8 ± 1.8
LDL	9.2 ± 0.4	10.9 ± 0.7	19.7 ± 3.2 **	12.2 ± 1.8 °
Triglycerides	100.8 ± 12.3	108.2 ± 10.9	139.3 ± 21.9	124.9 ± 11.6

Data are the mean ± SEM. ** *p* < 0.01 vs. CHOW rats, ° *p* <0.05 vs. CAF. CAF: cafeteria; P: probiotic supplementation; GOT: serum glutamic-oxaloacetic transaminase; GPT: serum glutamic pyruvic transaminase; GGT: gamma-glutamyltransferase; HDL: high-density lipoprotein; LDL: low-density lipoprotein.

**Table 2:** Leptin concentration in blood at the end of the experiment (day 84).

	CHOW Rats	CHOW+P Rats	CAF Rats	CAF+P Rats
Leptin concentration	2583.7 ± 557.4	2737.9 ± 276.3	5287.1 ± 773.4 *	1726.7 ± 265.1 °

Data, expressed as pg/mL, are the mean ± SEM. * *p* < 0.05 vs. CHOW, ° *p* < 0.05 vs. CAF. CAF: cafeteria; P: probiotic supplementation.

**Table 3:** Statistically significant correlations between the taxa relative abundances and variation of body weight and food intake in CAF and CAF+P rats.

	Family	Genus	Spearman *r*		*p* (Two-Tailed)
			Body Weight Gain (T84–T0)	Net Food Intake (T84–T0)	
CAF rats	*Propionibacteriaceae*	*Propionibacterium*	−0.6547	-	<0.0001
	*Porphyromonadaceae*	*Butyricimonas*	−0.9429	-	0.0167
	*Lachnospiraceae*	*Lachnospiraceae UCG-005*	−0.8857	-	0.0333
	*Ruminococcaceae*	*Subdoligranulum*	−0.3928	-	<0.0001
	*Corynebacteriaceae*	*Corynebacterium 1*	-	−0.8857	0.0333
	*Rikenellaceae*	*Rikenellaceae RC9 gut group*	-	−0.8857	0.0333
	*Ruminococcaceae*	*Faecalibacterium*	-	−0.9429	0.0167
CAF+P rats	*Coriobacteriaceae*	*Collinsella*	0.881	-	0.0072
	*Coriobacteriaceae*	*Coriobacteriaceae UCG-002*	−0.7619	-	0.0368
	*Lachnospiraceae*	*[Eubacterium] hallii group*	0.8095	-	0.0218
	*Ruminococcaceae*	*Ruminococcaceae UCG-008*	0.7619	-	0.0368
	*Ruminococcaceae*	*Subdoligranulum*	−0.2474	-	<0.0001
	*Lachnospiraceae*	*Lachnospiraceae NK4A136 group*	-	0.8333	0.0154
	*Lachnospiraceae*	*Marvinbryantia*	-	0.7857	0.0279
	*Peptostreptococcaceae*	*Intestinibacter*	-	−0.8095	0.0218
	*Peptostreptococcaceae*	*Peptoclostridium*	-	−0.8571	0.0107
	*Ruminococcaceae*	*Flavonifractor*	-	0.881	0.0072
	*Ruminococcaceae*	*Oscillospira*	-	0.7381	0.0458
	*Ruminococcaceae*	*Ruminiclostridium 6*	-	0.8333	0.0154
	*Erysipelotrichaceae*	*Turicibacter*	-	−0.8333	0.0154

**Table 4:** Total vancomycin- and gentamycin-resistant *Lactobacillus* count and recovery of *L. plantarum* IMC 510 in fecal samples of CAF+P rats at T0 and after 84 days of *L. plantarum* IMC 510 daily supplementation.

CAF+P Rats	T0	T84
*Lactobacillus* spp. (CFU/g of feces)	(5.0 ± 1.1) × 10^4^	(1.3 ± 1.0) × 10^5^
*L. plantarum* IMC 510 (CFU/g of feces)	0.0 ± 0.0	(7.8 ± 1.5) × 10^4^
Percentage of positive samples for the recovery of *L. plantarum* IMC 510	0%	100%

T0: before *Lactiplantibacillus (L.) plantarum* IMC 510 supplementation; T84: after 84 days of *L. plantarum* IMC 510 supplementation. CAF: cafeteria; P: probiotic supplementation.

## Data Availability

The data presented in this study are available on request from the corresponding author.

## Supplementary Materials

Available online at https://www.mdpi.com/article/10.3390/ijms222011171/s1.
