# Supplementary material for: Supplementation with Lactiplantibacillus plantarum IMC 510 Modifies Microbiota Composition and Prevents Body Weight Gain Induced by Cafeteria Diet in Rats
Source: Int J Mol Sci. 2021 Oct 16;22(20):11171. doi: 10.3390/ijms222011171 (PMC8540549; doi:10.3390/ijms222011171)
Supplement: Supplementary file 1 [file ijms-22-11171-s001.zip › ijms-1381143-supplementary.pdf]

Supporting Information

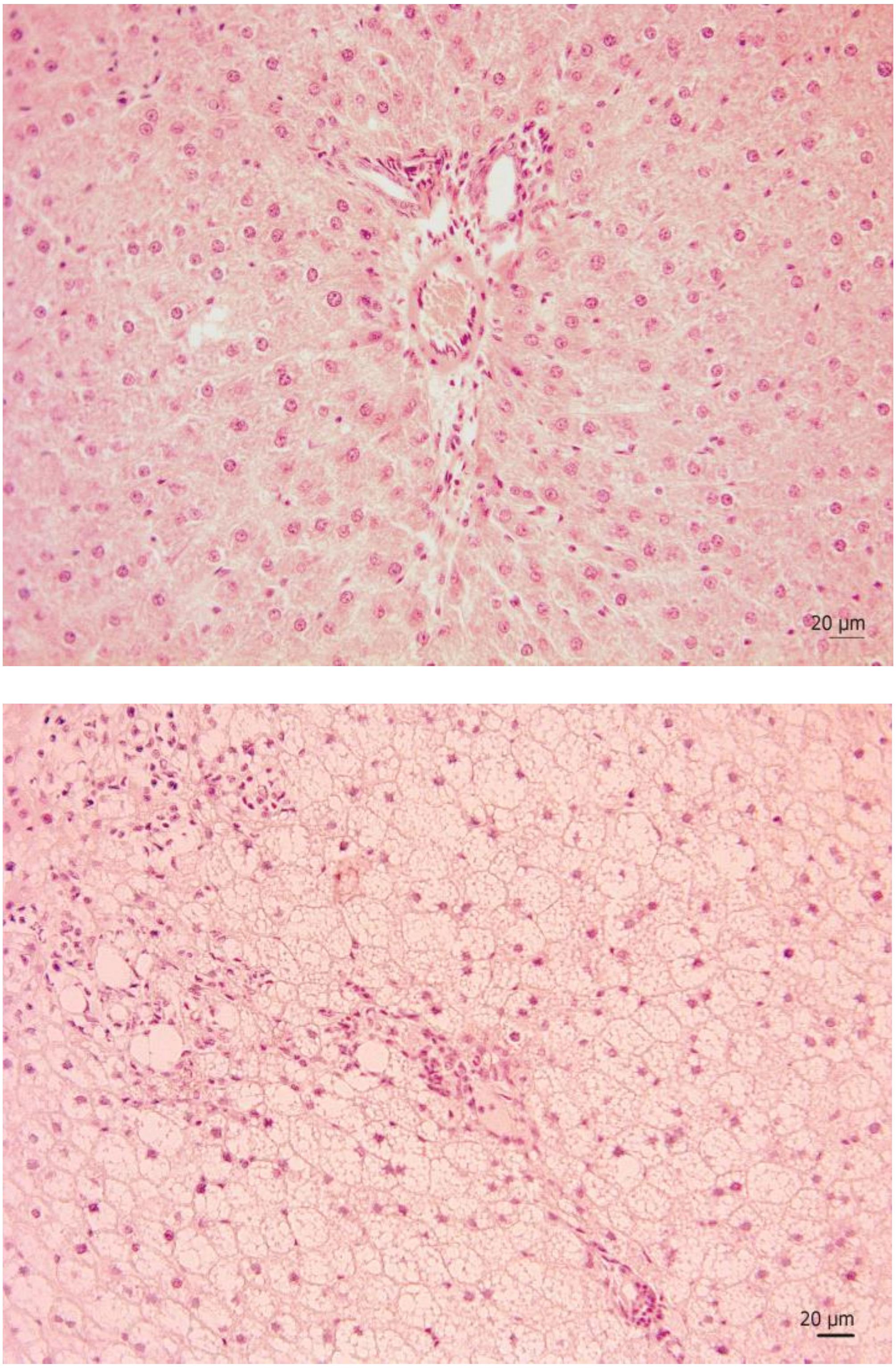

Figure S1. Hematoxylin and eosin (H& staining enhances the morphological features of liver from CHOW rats (and rats fed with cafeteria diet (in which wide areas of extensive microvesicular steatosis (and scattered macrovesicular steatosis arrow head is evident showing a preferential periportal localization Small infiltrating lymphocytes are evident (Calibration bar 20 mm).

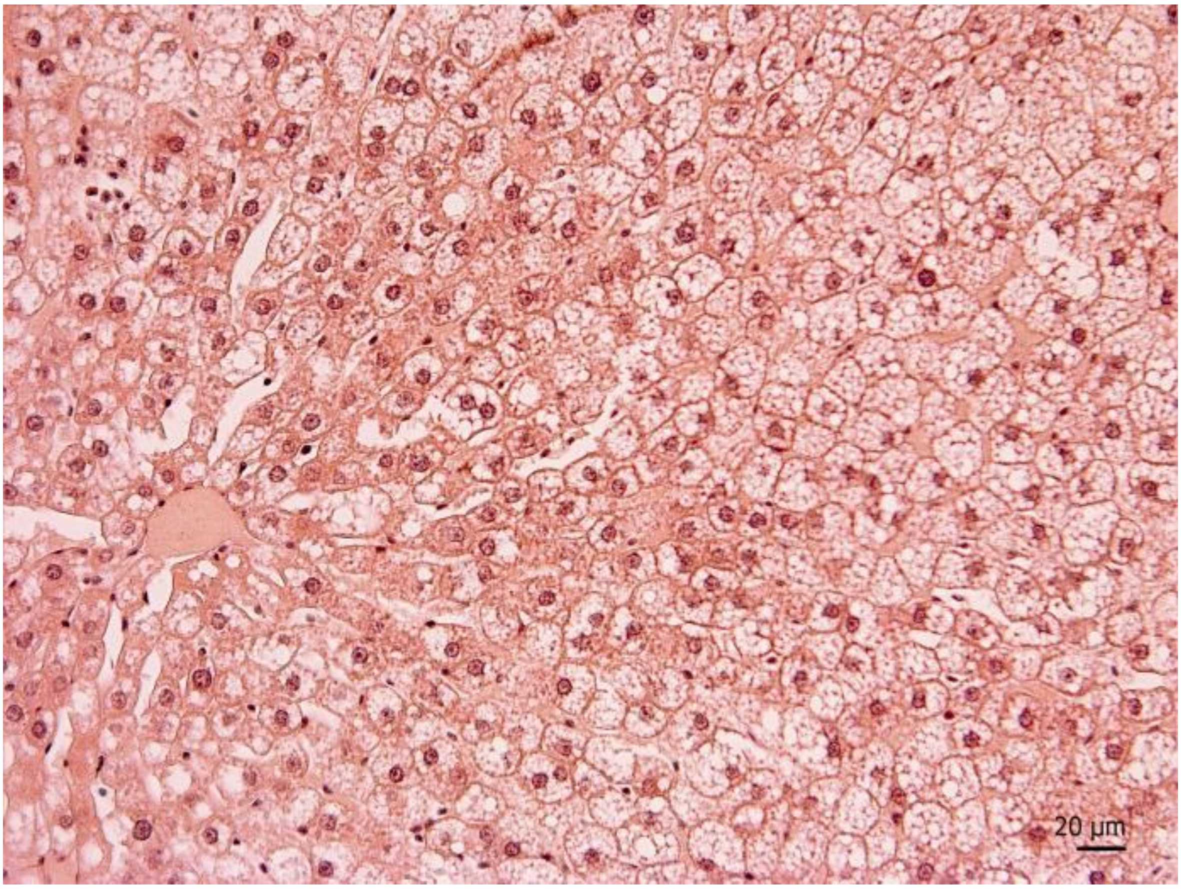

Figure S2. Hematoxylin and eosin (H& staining enhances the morphological features of liver from CHOW+P rats, in which wide areas of microvesicular steatosis (and scattered macrovesicular steatosis (arrow head) are evident Extension of the centrolobular zones with preserved hepatocytes are present Calibration bar 20 mm.
